# Monte Carlo Simulations of the GE Signa PET/MR for Different Radioisotopes

**DOI:** 10.3389/fphys.2020.525575

**Published:** 2020-09-15

**Authors:** Paulo R. R. V. Caribé, Stefaan Vandenberghe, André Diogo, David Pérez-Benito, Nikos Efthimiou, Charlotte Thyssen, Yves D’Asseler, Michel Koole

**Affiliations:** ^1^Medical Imaging and Signal Processing – MEDISIP, Ghent University, Ghent, Belgium; ^2^Faculty of Sciences of the University of Lisbon (FCUL), Lisbon, Portugal; ^3^Bioengineering and Aerospace Department, Universidad Carlos III de Madrid, Madrid, Spain; ^4^Department of Physics, University of York, York, United Kingdom; ^5^Department of Diagnostic Sciences, Faculty of Medicine, Ghent University, Ghent, Belgium; ^6^Nuclear Medicine and Molecular Imaging, Department of Imaging & Pathology, KU Leuven, Leuven, Belgium

**Keywords:** nuclear medicine, PET/MR, NEMA NU 2–2012, high energy positron emitters, positron range

## Abstract

NEMA characterization of PET systems is generally based on ^18^F because it is the most relevant radioisotope for the clinical use of PET. ^18^F has a half-life of 109.7 min and decays into stable ^18^O via β+ emission with a probability of over 96% and a maximum positron energy of 0.633 MeV. Other commercially available PET radioisotopes, such as ^82^Rb and ^68^Ga have more complex decay schemes with a variety of prompt gammas, which can directly fall into the energy window and induce false coincidence detections by the PET scanner.

**Methods:**

Aim of this work was three-fold: (A) Develop a GATE model of the GE Signa PET/MR to perform realistic and relevant Monte Carlo simulations (B) Validate this model with published sensitivity and Noise Equivalent Count Rate (NECR) data for ^18^F and ^68^Ga (C) Use the validated GATE-model to predict the system performance for other PET isotopes including ^11^C, ^15^O, ^13^N, ^82^Rb, and ^68^Ga and to evaluate the effect of a 3T magnetic field on the positron range.

**Results:**

Simulated sensitivity and NECR tests performed with the GATE-model for different radioisotopes were in line with literature values. Simulated sensitivities for ^18^F and ^68^Ga were 21.2 and 19.0/kBq, respectively, for the center position and 21.1 and 19.0 cps/kBq, respectively, for the 10 cm off-center position compared to the corresponding measured values of 21.8 and 20.0 cps/kBq for the center position and 21.1 and 19.6 cps/kBq for the 10 cm off-center position. In terms of NECR, the simulated peak NECR was 216.8 kcps at 17.40 kBq/ml for ^18^F and 207.1 kcps at 20.10 kBq/ml for ^68^Ga compared to the measured peak NECR of 216.8 kcps at 18.60 kBq/ml and 205.6 kcps at 20.40 kBq/ml for^18^F and ^68^Ga, respectively. For ^11^C, ^13^N, and ^15^O, results confirmed a peak NECR similar to ^18^F with the effective activity concentration scaled by the inverse of the positron fraction. For ^82^Rb, and ^68^Ga, the peak NECR was lower than for ^18^F while the corresponding activity concentrations were higher. For the higher energy positron emitters, the positron range was confirmed to be tissue-dependent with a reduction of the positron range by a factor of 3 to 4 in the plane perpendicular to the magnetic field and an increased positron range along the direction of the magnetic field.

**Conclusion:**

Monte-Carlo simulations were used to predict sensitivity and NECR performance of GE Signa PET/MR for ^18^F, ^15^O, ^13^N, ^11^C, ^82^Rb, and ^68^Ga radioisotopes and were in line with literature data. Simulations confirmed that sensitivity and NECR were influenced by the particular decay scheme of each isotope. As expected, the positron range decreased in the direction perpendicular to the 3T magnetic field. However, this will be only partially improving the resolution properties of a clinical PET/MR system due to the limiting spatial resolution of the PET detector.

## Introduction

The simultaneous acquisition of both PET and MRI was first developed for small animal imaging (Shao et al., 1997) whereas the development of an integrated PET/MR system for human studies only dated from a decade ago (Catana et al., 2010). The development of solid-state detectors such as avalanche photodiodes (APDs) and silicon photomultipliers (SiPMs) made it possible to integrate a PET detector ring into the bore of an MR scanner and to develop fully integrated PET/MR systems. The main advantage of using SiPMs over APDs is the faster detector response, therefore enabling simultaneous Time-of-Flight (TOF) PET and MR scanning, as demonstrated with the GE Signa PET/MR system (Yamamoto et al., 2003; Levin et al., 2016b; Vandenberghe et al., 2016; Efthimiou et al., 2019).

In terms of resolution, the PET spatial resolution is mainly limited by the detector size of discrete detector elements, the positron range, non-collinearity of the gamma rays resulting from an annihilation event and the decoding scheme of the PET scanner (Levin and Hoffman, 1999; Moses, 2011). The positron range, that is the distance the positron travels from the emitting nucleus to the location where the annihilation occurs, is the main non-detector related factor that limits the PET resolution (Moses, 1994; Palmer et al., 2005; Park et al., 2007; Peng and Levin, 2012) and the use of a magnetic field to limit its impact has already been proposed in the nineties (Iida et al., 1986; Rickey et al., 1992; Hammer et al., 1994; Laforest and Liu, 2008; Vallabhajosula et al., 2011). Because of the Lorentz force, a moving charged particle such as a positron describes a helical path along the direction of a magnetic field. As such, the positron range is reduced in the plane perpendicular to the magnetic field, while it remains unaltered or becomes slightly enlarged in the direction of the magnetic field (Eleftheriou et al., 2014; Kolb et al., 2015). Furthermore, the positron range relates directly to the energy of the positron (Kemerink et al., 2011; Emond et al., 2019). Studies with various PET radioisotopes have reported a larger reduction of the positron range for isotopes emitting positrons with a higher energy such as ^120^I (Herzog et al., 2010), ^82^Rb (Rahmim et al., 2008); or ^68^Rb (Wirrwar et al., 1997; Cal-González et al., 2009; Soultanidis et al., 2011; Alva-Sánchez et al., 2016; Li et al., 2017). In order to reduce the blurring effect of the positron range, it can be modeled as part of Point Spread Function (PSF), used in the reconstruction algorithm to model the PET system response. However, as stated in Jodal et al. (2012), different values for the positron range of different PET isotopes have been reported in literature, probably because of the limited accuracy of the experimental setup used in some of these studies such that the intrinsic detector resolution might be comparable to the positron range. As reference values for this study, we used the positron range values reported in Cal-González et al. (2009), Jodal et al. (2012) for the most relevant PET isotopes and various surrounding tissues.

Generally, the evaluation of the performance of a PET system is done for ^18^F because it is the most widely used PET isotope in a clinical setting to evaluate the glucose metabolism in mainly oncological but also specific cerebral (Phelps et al., 1979) and cardiac diseases (Marshall et al., 1983). However, with the increasing clinical relevance of other radioisotopes, such as ^15^O, ^13^N, ^11^C, ^82^Rb, and especially ^68^Ga (Hoffend et al., 2005), the need to evaluate the PET system performance for these isotopes is increasing because of their different decay scheme and physical properties. ^18^F almost exclusively decays via positron emission with a branching ratio of 96.8% and has a relatively low maximum positron energy (0.6335 MeV). Properties of ^15^O, ^13^N, and ^11^C, are in line with ^18^F properties and are considered pure β^+^ emitters, with the probability of positron emission being close to 100% and with a maximum positron energy of 1.735, 1.198, and 0.960 MeV, respectively. On the other hand, ^82^Rb and ^68^Ga have more complex decay schemes with multiple positron emission branches with different energies and with a significant contribution of prompt gamma emissions (Banerjee and Pomper, 2013; Afshar-Oromieh et al., 2014; Conti and Eriksson, 2016; Papp et al., 2018; Mayerhoefer et al., 2020). In addition, these PET radioisotopes emit positrons with a higher maximum energy of 3.381 and 1.8991 MeV, respectively (energy of the most abundant positron). In the context of simultaneous PET/MR imaging, the impact of a high magnetic field on positron range during PET scanning still needs extensive evaluation, especially for high-energy positron emitters.

Among the NEMA acceptance tests, the sensitivity and Noise Equivalent Count Rate (NECR) tests are essential to evaluate the PET system performance. Sensitivity expresses the fraction of coincidences resulting from β^+^ decay that is registered by the PET system for low activity concentrations. NECR is related to signal-to-noise ratio (SNR) and evaluates the impact of an increased activity concentration on the PET signal. It determines the interplay between true events, scatter and randoms to estimate which increase of activity concentration is still beneficial to improve the SNR.

The aim of this work was three-fold and can be summarized as follows: (A) Develop a realistic GATE model for the GE Signa PET/MR (B) Validate this model with sensitivity and NECR measurements performed on the 3T GE Signa PET/MR for ^18^F and ^68^Ga according to the NEMA NU 2–2012 protocol (National Electrical Manufacturers Association [NEMA], 2012; Caribé et al., 2019) (C) Use the validated GATE-model to predict the PET/MR system performance for other isotopes including ^18^F, ^11^C, ^15^O, ^13^N, ^82^Rb, and ^68^Ga and to evaluate the effect of the 3T magnetic field on the positron range.

## Materials and Methods

### GATE Model for the GE Signa PET/MR

GATE is a toolbox for Monte Carlo (MC) simulations based on GEANT4 and adapted for nuclear medicine applications (Jan et al., 2004). GATE Monte Carlo (MC) simulations were performed on a high-performance computer installed at Ghent University (Vlaams Supercomputer Centrum – VSC). The GATE model was implemented to mimic the PET hardware configuration of the integrated GE Signa PET/MR system consisting of five rings of 28 detector blocks each, covering an axial Field Of View (FOV) of 25 cm while the transaxial FOV is 60 cm. Each detector element consisted of a lutetium-yttrium-oxyorthosilicate (LYSO) scintillator with crystal elements of 25 mm × 4.0 mm × 5.3 mm and MR-compatible SiPM technology (Levin et al., 2016a). In addition, an energy window of 425–650 keV was used while the coincidence window was set to 4.57 ns (±2.29 ns). The geometry was modeled using the cylindrical PET system model in GATE, which also takes into account the foam, plastic and copper shielding between the bore and the detectors. This model was used to simulate the annihilation distribution of positrons for different tissue types and included the following physical processes: positron decay, multiple scattering, ionization, bremsstrahlung and electron annihilation. To evaluate the effect of a magnetic field on the positron range, a magnetic field with a field strength of 3.0 Tesla was in the axial direction to model the MR component of the GE Signa PET/MR system which has a static magnetic field of 3.0 Tesla with a maximum radiofrequency amplitude of 44 mT/m and a maximum slew rate of 200 T/m/s.

### Validation of the GATE Model for the GE Signa PET/MR

To validate the GATE model for the GE Signa PET/MR, we compared the sensitivity and NECR results of the NEMA NU 2–2012 acceptance measurements of the GE Signa PET/MR system using ^18^F and ^68^Ga with simulated sensitivity and NECR for ^18^F and ^68^Ga using GATE and the appropriate model for the GE Signa PET/MR and the hardware phantoms.

#### Sensitivity Measurements

Sensitivity measurements were performed at two different locations in the FOV according to the NEMA NU 2–2012 protocol (National Electrical Manufacturers Association [NEMA], 2012). At each location, multiple measurements were performed of a 700 mm long source filled with low levels of activity. The line source was surrounded by an aluminum cylinder of initially 2.5 mm thickness with successively adding four 2.5 mm thick aluminum sleeves. Low activity levels were used to minimize random events and dead time effects while the dense aluminum surroundings of the line source ensured sufficient annihilation events to measure the PET signal. To obtain sufficient count statistics, sensitivity data were measured as long as it took to have at least 10.000 true events collected per slice. The sensitivity was calculated by extrapolating the sensitivity values for the line source surrounded by an aluminum sleeve with varying thickness to the sensitivity value corresponding to the attenuation-free measurement of the line source via the following equation:

(1) $$S_{i}=S_{0}\times e^{-2\,\mu_{A\,l}\,X_{i}}$$

where *S*_*i*_ is the sensitivity corresponding to the *i*th measurement, *X*_*i*_ the thickness of the aluminum sleeve for the *i*th measurement, μ_*Al*_ the linear attenuation coefficient of aluminum and *S*_*0*_ actual sensitivity corresponding to a measurement with no aluminum surrounding the line source.

#### Sensitivity Simulations Using GATE

The simulations consisted of modeling a low activity line source filled with 5 MBq, positioned first in the center of the FOV and then at a radial distance of 10 cm from the center. To measure the sensitivity, simulations were performed of the line source surrounded by an aluminum sleeve with five different thicknesses ranging from 2.5 to 12.5 cm in steps of 2.5 cm. For each simulation, the count rate of true events only was obtained ROOT’s “Coincidences” tree which stores pairs of single events that meet the conditions specified in the digitizer. Each pair is identified by an *eventID* for each of the two single events (*eventID1* and *eventID2*), which identifies the radioactive decay generating the singles. Furthermore, the entire history of interactions, including Compton or Rayleigh scattering, occurring from their location of origin till they reach the detector, is recorded for each event of a pair. A coincidence detection is considered to be random when the *eventID* between the two single events of one pair is different. When they are identical, a coincidence event can still be either a scattered or true event. True coincidences are obtained by excluding paired events with a history of compton or rayleigh scattering. Corresponding sensitivity values were determined as described for the sensitivity measurements and the final sensitivity was reported as the average of both positions in the FOV.

#### NECR Measurements

Noise Equivalent Count Rate was measured with a 700 mm long and 203 mm wide polyethylene cylinder containing a 700 mm long plastic tube line source (3.2 mm inner diameter) filled with high activity. To evaluate the impact of random counts and dead time effects for different activity levels, the measurements were repeated to take advantage of the physical decay and cover different levels of activity. Through a sinogram-based analysis, as described below, the peak NECR, corresponding activity concentration and scatter fraction were extracted from these measurements (National Electrical Manufacturers Association [NEMA], 2012).

#### NECR Simulations Using GATE

For NECR simulations, the activity in the line source was varied from 1 to 800 MBq using a total of 11 different levels in order to reduce the computation time. In order to estimate the rate at which the scanner acquires coincidence data, that is *true*, *random*, or *scattered* coincidences, a sinogram based analysis was performed. For each slice, the sinogram stores the LOR as function of the projection angle and the distance from the center of the FOV. The GATE output file containing the sinogram data were imported as a 2D matrix and transformed into a 2D histogram with 320 bins for the vertical axis, representing the projection angle from 0 to π, and 640 projection bins for the horizontal axis, representing the distance from the center of the FOV ranging from −300 to 300 mm for the GE Signa PET/MR. All processing steps are shown in Figure 4.

**FIGURE 1:**
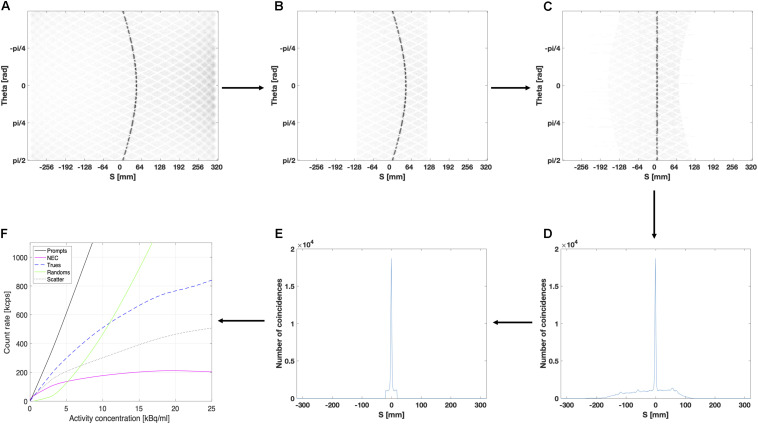
Extraction of NECR data: **(A)** Extract in ROOT the sinogram data for each slice, **(B)** Set all pixels located more than 12 cm from the center of the FOV to 0, **(C)** Align the projection bins of each projection angle according to the maximum values, **(D)** Sum all projection angles of the sinogram, **(E)** Select a 40 mm wide strip to estimate random and scattered events, **(F)** Compute the scatter fraction and different count rate curves as well as the NECR.

According to the NEMA protocol, an alignment of the sinogram data was performed by finding the maximum value for each projection angle and shifting the projection data such that the maximum value for each projection angle is at the center of the sinogram, as shown in Figures 1A–C. After alignment, the corresponding projection bins of all projection angles are summed to obtain a summed projection profile, as shown in Figure 1D. Next, a 40 mm wide strip (see Figure 1E) was centralized around the peak of the summed projection profile in order to estimate the background counts according to NEMA procedures. The values of the projection bins at left and right edge of this central 40 mm wide strip were averaged and multiplied by the number of projection bins within the strip. This value was considered as a representative estimate for the fraction of random and scattered events detected within the strip and used to estimate the corresponding fraction of true events. Once the fractions of random, scattered and true events are estimated for different activity levels, the corresponding count rate curves as well as NECR can be extracted (Figure 1F).

### Simulated Sensitivity and NECR of the GE Signa PET/MR for Other Isotopes Using GATE

The GATE-model for the GE Signa PET/MR was used to simulate the sensitivity and NECR of the PET/MR system for isotopes other than ^18^F and ^68^Ga including ^11^C, ^15^O, ^13^N, and ^82^Rb. In terms of sensitivity, the simulated values were compared with the theoretical sensitivities based the branching ratio of each isotope and the average sensitivity of 21.5 cps/kBq measured for ^18^F (Caribé et al., 2019).

### Positron Range Evaluation Using the GATE Model for the GE Signa PET/MR

The GATE-model for the GE Signa PET/MR was used to simulate the presence of the magnetic field and evaluate its impact on the positron range in different tissue media. To characterize the effect of the 3T magnetic field on the positron range, we simulated point sources of positron-emitting radionuclides including ^18^F, ^11^C, ^15^O, ^13^N, ^68^Ga, and ^82^Rb positioned in the middle of a homogeneous 20 cm × 20 cm × 20 cm cube with different tissue media: lung (mass density of 0.3 g/cm^3^), soft tissue (mass density of 1.0 g/cm^3^), bone (mass density of 1.42 g/cm^3^). To evaluate the tissue-dependence, we simulated the spatial distribution of positron annihilation for ^68^Ga in an inhomogeneous region. The region comprises two adjacent cubes of 20 cm × 20 cm × 20 cm filled with lung and soft tissue. For each isotope, 5 million events were simulated with and without 3T magnetic field applied in the axial direction. As output, the spatial coordinates of the location of the annihilation end point were saved to a file for each recorded positron.

## Results

### GATE Model for the GE Signa PET/MR

**FIGURE 2:**
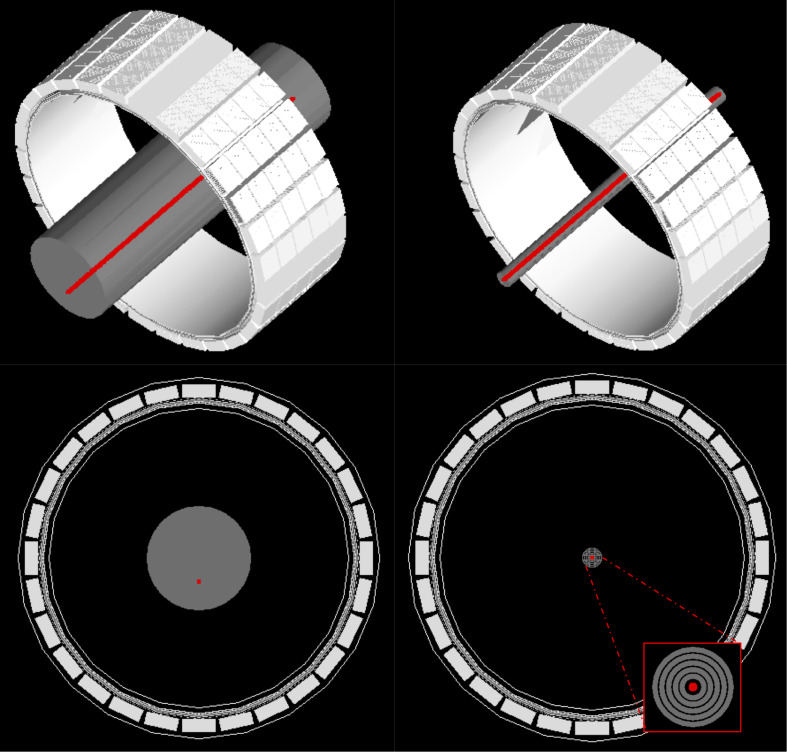
Visualization of the GATE model of the GE Signa PET/MR system including the NEMA NECR (right) and sensitivity (left) phantom with a 70-cm-line activity source (red). The PET system consists of 5 rings of 28 detector blocks (25 mm × 4.0 mm × 5.3 mm) based on lutetium-yttrium-oxyorthosilicate (LYSO) crystals with MR-compatible SiPM technology (Alva-Sánchez et al., 2016). This results in an axial and transaxial FOV of 25 and 60 cm, respectively.

The GATE model for the GE Signa PET/MR including the MR-body (gray) is presented in Figure 2 together with the phantom configurations for the sensitivity and NECR simulations according to the NEMA NU 2-2012 protocols.

### Validation of the GATE Model for the GE Signa PET/MR

The simulated and measured sensitivity values for ^18^F and ^68^Ga at the center of the FOV and 10 cm off-center are presented in Table 1 while the simulated and measured peak NECR with the corresponding activity concentration and scatter fraction at peak NECR are presented for ^18^F and ^68^Ga in Table 2.

**TABLE 1:** Sensitivity for ^18^F and ^68^Ga in the center of the FOV and 10 cm off center in the presence of 3T MR field.

	**Measured (cps/kBq)**		**Simulated (cps/kBq)**	
	**0 cm**	**10 cm**	**0 cm**	**10 cm**
^18^F	21.831	21.173	21.205	21.112
^68^Ga	20.063	19.689	19.098	19.017

*These results are compared to the measured ^ 18^F and ^68^Ga-values (Caribé et al., 2019).*

**TABLE 2:** Measured and simulated scatter fraction, peak NECR and the corresponding activity concentration for different isotopes in the presence of 3T magnetic field.

	**Scatter fraction at peak (%)**	**Peak NECR (kcps)**	**Activity concentration at peak NECR (kBq/ml)**
**Measured**			
^18^F (Caribé et al., 2019)	43.3	216.8	18.60
^18^Ga (Caribé et al., 2019)	42.9	205.6	20.40
**Simulated**			
^18^F	38.8	216.8	17.4
^15^O	38.8	216.4	18.2
^13^N	38.2	212.0	16.5
^011^C	38.5	217.6	16.7
^82^Rb	39.1	173.5	19.6
^68^Ga	38.7	207.1	20.1

### Simulated Sensitivity and NECR of the GE Signa PET/MR for Other Isotopes Using GATE

**FIGURE 3:**
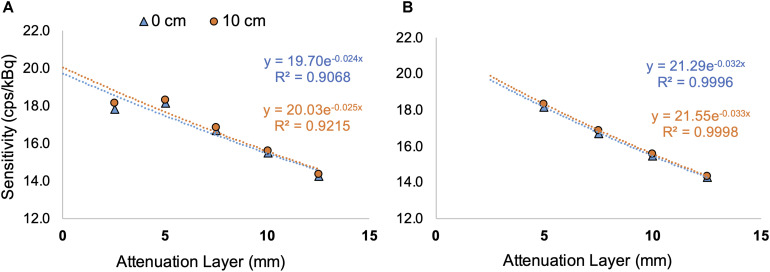
Sensitivity data for ^82^Rb with the sensitivity plotted against the thickness of the attenuation layer together with the exponential regression. Graphs show the fitted equation and coefficient of determination for the simulations at the center of the FOV (0 cm, blue) and for 10 cm radially off center (10 cm, orange) with, respectively, 5 **(A)** and 4 **(B)** different thicknesses for the attenuation layers.

Table 3 shows the simulated NEMA sensitivity for ^18^F, ^15^O, ^13^N, ^82^Rb, and ^68^Ga as the average value of the sensitivity at the center of the FOV and 10 cm off-center. Figure 3A shows the underestimation of the simulated sensitivity for ^82^Rb when only one aluminum sleeve is surrounding the line source while Figure 3B presents the estimated sensitivity without taken into account the sensitivity values using only one aluminum sleeve. As such, the sensitivity value averaged over the center and 10 cm off center position in the FOV is increased to 21.4 cps/kBq (Figure 3B), compared to 19.9 cps/kBq when taken into account all five sleeve thicknesses (Figure 3A).

**TABLE 3:** Sensitivity for different isotopes in the presence of 3T magnetic field, presented as the average value of the sensitivity at the center of the FOV and the sensitivity at 10 cm off center.

	**Branching ratio (%)**	**Measured (cps/kBq) (Caribé et al., 2019)**	**Simulated (cps/kBq)**	**Theoretical values (cps/kBq)**	***R*^2^ at the center values**
^18^F	96.76	21.5	21.2		1.00
^15^O	99.89		20.9	22.20	1.00
^13^N	99.82		21.5	22.18	1.00
^11^C	99.75		21.1	22.16	1.00
^82^Rb	95.45		19.9	21.21	0.91
^68^Ga	87.90	19.9	19.2	19.53	1.00

**FIGURE 4:**
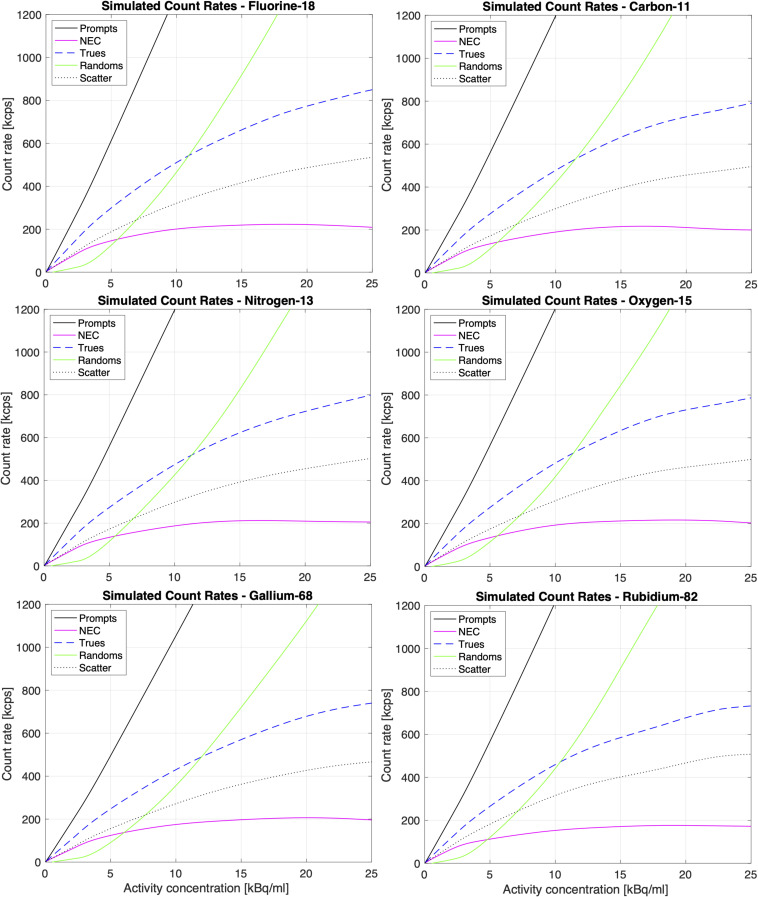
Simulated count rates and NECR for ^18^F, ^15^O, ^11^C, ^13^N, ^82^Rb, and ^68^Ga according to NEMA, as a function of the activity concentration.

The results for the simulated peak NECR, the corresponding activity concentration and the scatter fraction at peak NECR for ^18^F, ^11^C, ^15^O, ^13^N, ^68^Ga, and ^82^Rb are presented in Table 2 while Figure 4 shows the simulated true, random and scattered coincidence rates as well as NECR curves for these isotopes as a function of the activity concentration.

### Positron Range Evaluation Using the GATE Model for the GE Signa PET/MR

**FIGURE 5:**
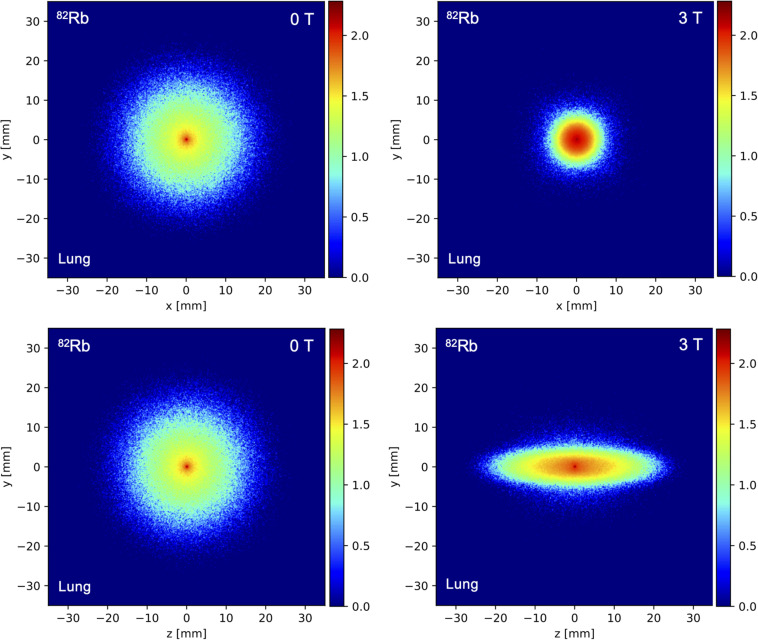
Spatial distribution of the simulated annihilation endpoints in the *x*/*y* plane perpendicular to the magnetic field and the *z*/*y* plane parallel to the magnetic field for a ^82^Rb point source positioned in homogeneous lung tissue for a field strength of 0 T (left) and 3 T (right).

**FIGURE 6:**
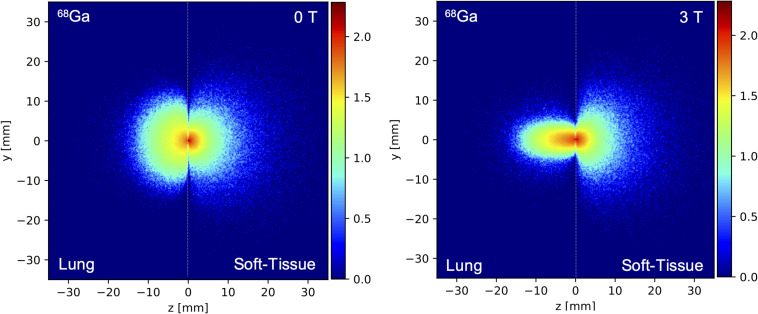
Spatial distribution of the simulated annihilation endpoints in the *z*/*y* plane parallel to the magnetic field for a ^68^Ga point source positioned at the interface between lung and soft tissue (dashed black line) for a field strength of 0 T (left) and 3 T (right).

Table 4 shows the comparison of the simulated positron range in soft tissue for different radioisotopes with and without 3T magnetic field with measured values taken from literature (Li et al., 2017; Soultanidis et al., 2011). The simulated mean positron range values for different isotopes in soft tissue, lung and bone are presented on Tables 5, 6 with and without the presence of a 3T magnetic field, with the mean positron range averaged over all directions (Table 5) and the mean positron range calculated for the transversal plane perpendicular to the magnetic field and axial plane parallel to the magnetic field (Table 6). The impact of the magnetic field on the positron range is also visually shown in Figures 5, 6.

**TABLE 4:** Comparison of positron range in soft tissue for different radioisotopes with and without 3T magnetic field.

	**Max energy (keV)**	**Mean 3D positron range (mm)**					
		**GATE**		**Ref.**		**Ref.**	
				**(Li et al., 2017)**		**(Soultanidis et al., 2011)**	
				
		**None**	**3T**	**None**	**3T**	**None**	**3T**
^18^F	633.5	0.50	0.52	0.64	0.45	0.56	0.54
^15^O	1732.0	1.87	1.66	2.01	1.74	2.44	2.00
^13^N	1198.5	1.08	1.01	1.32	1.26		
^11^C	960.2	1.02	0.96	1.03	0.82	1.05	0.96
^82^Rb	3378.0	4.85	3.82	4.29	3.65	5.21	3.90
^68^Ga	1899.0	2.32	2.04	2.24	2.02	2.62	2.07

**TABLE 5:** Mean 3D positron range for different tissues and radioisotopes with and without 3T magnetic field.

	**Mean 3D positron range (mm)**					
	**Soft tissue**		**Lung**		**Bone**	
			
	**None**	**3T**	**None**	**3T**	**None**	**3T**
^18^F	0.50	0.52	2.23	1.70	0.34	0.34
^15^O	1.87	1.66	7.74	4.28	1.22	1.17
^13^N	1.08	1.01	4.30	2.63	0.71	0.69
^11^C	1.02	0.96	3.05	1.97	0.51	0.51
^82^Rb	4.85	3.82	18.2	10.1	3.09	2.74
^68^Ga	2.32	2.04	8.09	4.59	1.33	1.26

**TABLE 6:** Mean positron range in the transversal (perpendicular to the magnetic field) and axial direction (parallel to the magnetic field) for different tissue types and radioisotopes with and without 3T magnetic field.

	**Mean transversal positron**						**Mean axial positron**					
	**range (mm)**						**range (mm)**					
	**Soft tissue**		**Lung**		**Bone**		**Soft tissue**		**Lung**		**Bone**	
						
	**None**	**3T**	**None**	**3T**	**None**	**3T**	**None**	**3T**	**None**	**3T**	**None**	**3T**
^18^F	0.27	0.26	0.95	0.73	0.17	0.17	0.27	0.27	0.95	1.08	0.17	0.17
^15^O	0.93	0.77	3.74	0.97	0.61	0.57	0.93	0.93	3.75	3.74	0.61	0.61
^13^N	0.54	0.49	2.15	0.74	0.35	0.34	0.54	0.54	2.15	2.15	0.35	0.35
^11^C	0.48	0.48	1.52	0.63	0.26	0.25	0.48	0.51	1.52	1.52	0.26	0.26
^82^Rb	2.42	1.62	9.10	2.25	1.54	1.28	2.42	2.42	8.95	8.96	1.54	1.55
^68^Ga	1.01	0.95	4.04	1.00	0.66	0.61	1.01	1.16	4.05	4.04	0.66	0.66

## Discussion

In this work we have developed and evaluated a model for the GE Signa PET/MR to run realistic Monte Carlo simulations using GATE. The model was validated by comparing simulated sensitivity and NECR data ^18^F and ^68^Ga with corresponding measurements performed on a GE Signa PET/MR system (Caribé et al., 2019). Once validated, these simulations allowed us to evaluate the system characteristics in terms of sensitivity and NECR for different, less conventional PET isotopes such as ^15^O, ^13^N, ^11^C, and ^82^Rb. In addition, the effect of the 3T magnetic field on positron range was investigated for different PET isotopes in different tissue types (lung tissue, soft tissue, and bone).

In terms of validating the GATE MC simulations, the simulated NEMA sensitivity values, presented in Tables 1, 3, were in line with the expected, theoretical values for all simulated PET radioisotopes, These theoretical values were based on the measured GE Signa PET/MR sensitivity for ^18^F while accounting for the differences in branching ratio between each isotope and ^18^F. These sensitivity values confirmed the higher count rate and increased sensitivity (Iagaru et al., 2019) of the GE Signa PET system due several design factors including Compton scatter recovery, longer axial FOV and reduced ring diameter (Lubberink and Herzog, 2011; Wagadarikar et al., 2014; Hsu et al., 2017).

For the pure β^+^ emitters such as ^11^C, ^13^N, and ^15^O, the sensitivity was comparable to that of ^18^F. However, ^68^Ga and ^82^Rb showed considerable differences. For ^68^Ga, this was expected as literature data already reported a sensitivity of about 2 cps/kBq lower than ^18^F (Peng and Levin, 2012), which was confirmed by the simulations. However, for ^82^Rb with a positron branching ratio very similar to ^18^F (less than 2% difference), the sensitivity is much lower than expected. This could be explained by the high energy of 3.381 MeV of the emitted positrons such that only one aluminum sleeve surrounding the line source is not adequate enough to generate sufficient annihilations. Therefore, sensitivity measurements with only one attenuating layer of aluminum surrounding the line source should be discarded for ^82^Rb or material with a higher density than aluminum should be considered (see Figure 3 and Table 3). In addition, and specifically for ^82^Rb, a significant portion of the coincidences were detected outside of the scanner bore, which, in theory, is not be possible since the LOR corresponding to two annihilation photons detected by the scanner can be positioned outside of the scanner. However, due to the additional 777 keV prompt-gamma emission, two gamma photons originating from a decaying ^82^Rb source, can be registered as a pair of annihilation photons by the scanner, even when the source is situated outside of the scanner bore.

The results of the simulated NEMA count rate performance tests of the GE Signa PET/MR are summarized in Figure 4 and Table 2 and showed good agreement with previous count rate data for ^18^F (Levin et al., 2016a). These results confirmed that GATE MC simulations can be used to study the count rate performance of the GE Signa PET/MR. For positron emitters with high branching ratios for β^+^ decay such as ^11^C, ^13^N, and ^15^O, the simulation results confirmed a peak NECR similar to ^18^F with the corresponding activity concentration scaled by the inverse of the positron fraction.

For the higher energy positron emitters such as ^68^Ga and ^82^Rb, the simulated count rates were slightly lower than the measured values for ^18^F, as shown in Figure 4 and Table 2. These lower values are primarily due to the respective 1.2% (1.883 MeV) and 13.1% (2.604 MeV) fraction of β^+^ decay for ^68^Ga and ^82^Rb, which also results in prompt gamma emissions. These prompt gammas contaminate the PET signal by generating additional random, scattered and detection events which, in turn, increases the deadtime effects. The third gamma effect was also reported by different research groups (Martin et al., 1995; Converse et al., 2003).

In terms of the positron range, the 3T magnetic field clearly reduced the positron range by a factor up to 3–4 in the direction perpendicular to the magnetic field, especially for the higher energy positron emitters such as ^15^O, ^68^Ga, and ^82^Rb and for low-density tissues such as lung tissue. While changes in positron range could clearly be observed in the perpendicular direction, the magnetic field does not have a clear impact on the positron range in the direction of the magnetic field, as was shown in Figure 5 and Tables 4–6. The impact of the magnetic field on the positron range is highly dependent on the positron energy and tissue type as shown in Tables 5, 6. The latter is known to be related to the tissue electron density such that the mean free path of the positron is larger for tissues with lower electron density (Cal-González et al., 2013). This dependency is clearly demonstrated at the interface between different tissues as shown in Figure 6. These findings are in agreement with previous studies on the positron range (Rickey et al., 1992; Cal-González et al., 2009; Shah et al., 2014; Alva-Sánchez et al., 2016; Huang et al., 2016; Caribé et al., 2017), and confirm a reduced positron range in the perpendicular direction of a magnetic field. Moreover, studies have also indicated (Iida et al., 1986; Wirrwar et al., 1997; Soultanidis et al., 2011; Kraus et al., 2012) that a higher magnetic field will also induce a greater reduction of the positron range. However, it has to be noted that this effect was negligible for low energy positron emitters, such as ^18^F. Moreover, the expected improvement of the PET image resolution, resulting from a reduced positron range by the presence of a 3T magnetic field, will only partially be observed in the resolution properties of the GE Signa PET/MR system due to the limited resolution of the PET detectors. Indeed, detector-dependent factors, such as crystal size and crystal penetration during detection, and inherent limitations such as the non-collinearity of the annihilation photons are also present and can explain why a reduced positron range with an increasing magnetic field does not translate directly into improved image quality (Herzog et al., 2010; Bertolli et al., 2016; Caribé et al., 2019; Wadhwa et al., 2020). However, in a preclinical setting with small diameter detector rings and crystal sizes, the impact of a reduced positron range on the PET image quality is expected to be much higher while the use of monolithic crystals or recordings of the depth of interaction in clinical PET systems can further enhance the PET resolution such that it becomes more sensitive to positron range effects (Hammer et al., 1994; Stockhoff et al., 2019).

However, there are also limitations to be considered for this study. The dead time digitizer settings have a certain degree of uncertainty since these values were not provide by the manufacturer. This could affect the NEMA sensitivity estimates via the exponential regression of the simulated data (Khalif et al., 2016) and could explain the slightly lower simulated sensitivity values compared to theoretical values. Due to the uncertainty of the dead time digitizer settings, the deadtime was heuristically tuned to match measured data, and then applied to other simulations. These settings could also have lowered the scatter count rate (∼500 kcps scatter at 20 kBq/ml as shown in Figure 4), compared to ∼700 kcps (Levin et al., 2016a) and ∼600 kcps (Caribé et al., 2019) scatter (at the same activity concentration) reported values for the Signa PET/MR with ^18^F. Moreover, it should be noted that the count rates and NECRs for each simulation in this study were estimated using the NEMA approach which does not require an estimate for the number of random events (National Electrical Manufacturers Association [NEMA], 2012). This could explain the slight underestimation of the scatter fraction which was determined directly from the count events in the ROOT file. Indeed, for all of isotopes, the simulated scatter fractions were around 38 to 40%, which is slightly lower compared to reported, measured scatter fraction of 43.3% for ^18^F. Finally, this study only evaluated the impact of a 3T magnetic field on the positron range, as this is a field strength which is clinically relevant and in line with the magnetic field of GE Signa PET/MR.

## Conclusion

GATE Monte-Carlo simulations were validated for simulating the GE Signa PET/MR system and used to predict sensitivity and NECR performance for ^18^F, ^15^O, ^13^N, ^11^C, ^82^Rb, and ^68^Ga. The GATE based predicted sensitivity and NECR data were in line with expected and previously published sensitivity and NECR values for all simulated PET isotopes and confirmed the impact of deadtime effects and increased random events on NECR for ^68^Ga and ^82^Rb because of the additional prompt gamma emissions. In addition, we have investigated the impact of the magnetic field on the positron range for different tissue types and PET isotopes. The improvement of image resolution resulting from a decreased positron range in the plane perpendicular to a 3T magnetic field, especially for high energy positron emitters, is only partially observed in the resolution properties of the GE Signa PET/MR system due to the limited spatial resolution of the PET detectors.

## Data Availability Statement

The datasets generated for this study are available on request to the corresponding author.

## Author Contributions

PC performed the conceptualization. PC, AD, NE, and CT contributed to the simulations of the study. PC and AD performed the data collection. PC, AD, and DP-B completed the data analysis. All authors discussed the results and commented on the manuscript, contributed to the design of the study, and read and approved the final manuscript.

## Conflict of Interest

The authors declare that the research was conducted in the absence of any commercial or financial relationships that could be construed as a potential conflict of interest.
